# 

**Published:** 1900-06-23

**Authors:** 


NOTICE TO CORRESPONDENTS.

All MS., Letters, Books for Review, and other matters intended for the Editor should be addressed The Editor, "The Hospital," The Hospital Building, 28 & 29, Southampton Street, Strand, W.C.

The Editor cannot undertake to return rejected MS., even when accompanied by stamped directed envelope.

**Notice to Advertisers and Subscribers.**

All Advertisements, Orders for Copies of The Hospital, and Business Communications should be addressed to **THE MANAGER (Not to the Editor)**, The Hospital, The Hospital Building, 28 & 29, Southampton Street, Strand, London, W.C.

The Cost of Subscription, post free, is as follows:—Per week, 2½d.; per quarter, 2s. 8d.; per half-year, 5s. 3d.; per annum, 10s. 6d. Foreign:—Per annum, 15s. 2d., payable, in all cases, in advance.

**NOTICE.—Vol. XXVII. of "The Hospital" (October, 1899, to April, 1900), in handsome cloth binding, is now on sale at the Office of this Journal, price 7s. 6d. Cases for binding the Numbers contained in this Volume 1s. 6d. Index to Vol. XXVII., 2½d., post free.**

**The Monthly Part for July will be ready on July 2nd. Price 6d., by post 8d.**

### Scraps and Gleanings.

The new cottage hospital at Colne, which contains accommodation for 14 patients, has cost over £4,000.

The recent subscription dance in aid of the Victoria Hospital for Nervous Diseases, Belfast, produced £50.

The result of the first day's takings at the bazaar on behalf of the Boscombe Hospital Building Fund was over £300.

A testimonial has recently been presented to Dr. Bruce, resident house surgeon at the Victoria Jubilee Hospital, Tynemouth.

Messrs. Brown and Polson have made a second contribution to the Indian Famine Fund, the donation on this occasion taking the form of 2,000 tins of their corn flour.

### THE NURSE'S REPORT BOOK.
Foolscap 4to., Black cover, **6d.** net. By Post 8d.

**Compiled by K. H. (Cert.)**

H. J. Glaisher, 57, Wigmore Street, Cavendish Square, W.

A REPRINT OF

### A NURSING HANDBOOK

TOGETHER WITH S. MAW, SON, AND THOMPSON'S

### COMPLETE CATALOGUE OF NURSING REQUISITES.

The above work is now republished at a cost of 1s. per copy. Messrs. S. M., S., and T. have been compelled to make this charge on account of the extreme popularity of the work and the great expense incurred in its production.

***Post Free on receipt of 1s.***

### S. MAW, SON, and THOMPSON,

7 to 12, ALDERSGATE STREET, LONDON, E.C.

